# *Chlamydia trachomatis* Serovars from the C-Complex and the B- and C-Related Complexes Are Significantly More Pathogenic than Those from the B-Complex in C3H/HeN but Not in BALB/c Mice

**DOI:** 10.3390/pathogens14010097

**Published:** 2025-01-19

**Authors:** Sukumar Pal, Jennifer R. Carmichael, Delia F. Tifrea, Olga Tatarenkova, Luis M. de la Maza

**Affiliations:** Department of Pathology and Laboratory Medicine, Medical Sciences I, Room D440, University of California, Irvine, Irvine, CA 92697-4800, USA; spal@exchange.uci.edu (S.P.); jcarmich@sdccd.edu (J.R.C.); dtifrea@uci.edu (D.F.T.); otataren@uci.edu (O.T.)

**Keywords:** *Chlamydia trachomatis*, serovars, mice, genital infection, vaginal shedding, infertility, upper genital tract pathology, genetic background

## Abstract

Studies in humans indicate that certain *Chlamydia trachomatis* serovars are more pathogenic than others. Specifically, several studies concluded that serovars from the C-complex are more pathogenic than those from the B-complex, although there are reports that do not support this finding. To investigate these results in an animal model, the eight genitourinary *C. trachomatis* serovars were tested in two strains of mice: C3H/HeN and BALB/c. These two strains of mice were investigated because C3H/HeN is more susceptible to *Chlamydia muridarum* infections than BALB/c, indicative of differences in their immunogenetic background. Mice were infected transcervically with 10^5^ inclusion forming units of each of the *C. trachomatis* serovars, and vaginal cultures were collected. To determine the pathogenicity and its impact on fertility, at week seven post-infection, female mice were caged with male mice. In the C3H/HeN mice, significant differences in vaginal shedding and fertility were observed between serovars from the B-complex (D and E) and those from the C-complex (H, I, J) and B- and C-related complexes (G, F, and K). The animals infected with serovars F, G, H, I, J, and K shed less but had significantly more infertility than the mice infected with serovars D or E. The experiments in the BALB/c mice, however, did not show major differences in pathogenicity between the eight *C. trachomatis* serovars. These results support the findings in humans and emphasize the critical importance of the immunogenetic background of the host on the outcome of *C. trachomatis* infections. The data imply that management of *C. trachomatis*-infected patients may require a more personalized approach.

## 1. Introduction

*Chlamydia trachomatis* is the most frequently isolated sexually transmitted bacterial pathogen worldwide [1,2]. *C. trachomatis* also produces ocular, respiratory, and gastrointestinal infections with a wide range of clinical presentations [3]. In women, most of the genital infections are asymptomatic [3]. However, in some patients, acute cervicitis and urethritis and long-term sequelae, including pelvic inflammatory diseases (PID), chronic abdominal pain, ectopic pregnancy, and infertility, occur [4]. Based on an analysis in the United Kingdom, Price et al. [5] concluded that, for every 1000 *C. trachomatis* infections in women aged 16–44 years, there are, on average, 171 episodes of PID, 73 episodes of salpingitis, 2 episodes of ectopic pregnancy, and 5.1 women with infertility at age 44 years. Understanding the host and pathogen characteristics that lead to these different outcomes is necessary if we want to implement effective preventative and therapeutic interventions.

Following the isolation of multiple human isolates of *C. trachomatis*, Wang and Grayston [6] performed cross-protection experiments in a mouse model. Mice immunized with one *C. trachomatis* strain were subsequently challenged with homologous and heterologous isolates. The degree of protection was quantified, and a relationship was established between all the *C. trachomatis* isolates. This interrelation between strains was further supported by cross-reacting sera from mice inoculated intravenously with the *C. trachomatis* isolates [7]. The results of the two experiments led to the division of the *C. trachomatis* human isolates into 15 major immunotypes/serovars [7]. The immunotypes were found to fall into two major groups: the B-complex and the C-complex. The C-complex includes the C, J, H, I, and A immunotypes, and the B-complex contains serovars B, Ba, E, D, L1, and L2. Types G and F are related to the B-complex, while types K and L3 are related to the C-complex. Serovars A, B, Ba, and C cause mainly trachoma; D-K cause mainly genitourinary infections; and the L1, L2 and L3 serovars are the etiological agents of lymphogranuloma venereum, a disease that mainly affects the reticuloendothelial system of the genitourinary and gastrointestinal tracts [3]. When the DNA sequence of *C. trachomatis* was determined, the serovar classification was found to correlate with the sequence of the major outer membrane protein (MOMP), specifically to the four variable domains (VD) that are thought to be surface exposed and, therefore, under immunological pressure [8]. Phylogenetic analysis of the MOMP DNA sequence from all the *C. trachomatis* serovars correlated with the classification established by Wang and Grayston [6,8]. Among the eight genitourinary serovars, E, D, and F are the most frequently recovered from clinical specimens [9,10,11].

Several human studies have correlated the severity of the clinical presentation with *C. trachomatis* serovars from the C- and the B-related complexes [12,13,14,15,16,17,18,19,20,21,22,23,24,25]. For example, in a study that included 2927 women, Hollegaard et al. [14] found a significant association between individuals infected with *C. trachomatis* C-complex serovars and the risk of preterm birth. In addition, they observed that women positive for *C. trachomatis* C-complex serovars were at a higher risk of being subfertile, having in the past been treated for infertility or ectopic pregnancy. Among 135 women attending a sexually transmitted infections (STI) clinic, van Duynhoven et al. [16] determined that the F and G serovars induced more lower abdominal pain than the other *C. trachomatis* serovars. In a study that included 226 female sex workers, Gao et al. [17] reported that, while serovar G was associated with lower abdominal pain, serovar E was found in 47.5% of asymptomatic patients. In a large retrospective study, Anttila et al. [26] reported that serovar G was the most strongly associated with cervical squamous cell carcinoma. Among 480 women with a *C. trachomatis* infection, Geisler et al. [19] found that those having abdominal pain and/or dyspareunia were more often infected with serovar F. Morre et al. [22] studied 230 women with asymptomatic and 100 women with symptomatic *C. trachomatis* infections. The three most common serovars identified, D, E, and F, showed no relationship with symptomatic or asymptomatic infections. Serovar K, however, was associated with vaginal discharge. Similarly, Dean et al. [25] studied a group of 552 women with more than three *C. trachomatis* recurrences over a 2-year period. Among them, 130 women had same serovar recurrences, including 58 that belonged to the C-complex. Seven women with three to ten repeated same serovar infections over a 2–5-year period were studied. Of these patients, only one was infected with a serovar D isolate, while the other six were positive with serovars from the C-complex. Based on these results, the authors concluded that serovars from the C-complex are the most likely to produce persistent infections and that MOMP may have specific biological properties that modulate the responses to immune selection.

In contrast to these findings, a study by van de Laar et al. [21] that included 116 women attending an STI clinic found no correlation between serovars and clinical manifestations. Batteiger et al. [12] evaluated the relationship between acute inflammation and *C. trachomatis* serovars in a group of individuals attending an STI clinic. The distribution of serovars in 99 women did not differ based on the absence or presence of cervicitis. Millman et al. [23] analyzed *C. trachomatis* isolates from 164 women and concluded that there was no association between serovars and clinical phenotypes. Workowski et al. [13] studied 99 women with lower genital tract infections and 56 with PID. Serovar F was associated with less clinical manifestations of mucopurulent endocervical discharge, while there was no association of PID with any specific serovar.

To address these apparently contradictory results in humans, we tested the ability of the eight genitourinary *C. trachomatis* serovars to produce genital tract pathology and infertility in two strains of mice. We used C3H/HeN mice that are highly susceptible to genital infections with *Chlamydia muridarum* (previously called *C. trachomatis* mouse pneumonitis biovar) and BALB/c mice that are more resistant [27,28]. To our knowledge, this is the first time that the eight *C. trachomatis* genitourinary serovars have been tested for their ability to infect and to produce pathology and infertility in two strains of mice with different genetic backgrounds. Our results in C3H/HeN mice support the evidence in humans that C-complex and the B- and C-related complexes serovars are significantly more pathogenic than those from the B-complex but shed less IFU. In contrast, no major differences in pathogenicity were found when infecting BALB/c mice, backing studies that found no differences in the pathogenicity among the *C. trachomatis* serovars. These findings indicate that the diversity in pathogenicity of the *C. trachomatis* serovars is manifested depending on the immunogenetic background of the host and suggest that the management of patients may require a more personalized approach.

## 2. Materials and Methods

### 2.1. C. trachomatis Stocks

The *C. trachomatis* serovars D (UW-3/Cx), E (Bour), F (IC-Cal-3), G (UW-57/Cx), H (UW-43/Cx), I (UW-12/Ur), J (UW-36/Cx), and K (UW-31-Cx) were purchased from the American Type Culture Collection (ATCC; Manassas, VA, USA). *Chlamydiae* stocks were grown in HeLa-229 cells (ATCC) using centrifugation as described [29]. Elementary bodies (EB) were purified and stored at −80 °C in 0.2 M sucrose, 20 mM sodium phosphate (pH 7.4), and 5 mM glutamic acid (SPG), as reported by Caldwell et al. [29]. The number of *C. trachomatis* inclusion forming units (IFU) of the stocks was determined in HeLa-229 cell cultures [29].

### 2.2. C. trachomatis Transcervical Infection of C3H/HeN (H-2^k^) and BALB/c (H-2^d^) Mice

Ten-to-11-week-old female mice, purchased from Charles River Laboratories (Wilmington, MA, USA), were housed at the University of California, Irvine, Vivarium. The animal protocols were approved by the UCI Animal Care and Use Committee. All experiments were replicated once.

To synchronize the estrus cycle in diestrus, four days before infection, mice were injected subcutaneously with 2 mg/mouse of medroxyprogesterone acetate (MPA) (Greenstone Ltd., Peapak, NJ, USA). The diestrus phase of the estrus cycle was confirmed by vaginal cytology [30]. For transcervical (t.c.) infection, mice were anesthetized, and an inoculum of 1 × 10^5^ IFU of the *C. trachomatis* serovars was delivered via the ox cervix by using a non-surgical embryo transfer device (ParaTechs Corp. Lexington, KY, USA) [31]. As controls, a group of mice was inoculated t.c. with SPG, and a fertility control group was not inoculated.

#### 2.2.1. Vaginal Cultures Collection

Following the genital infection, vaginal cultures were collected twice a week for the first four weeks and then at 7-day intervals for two additional weeks [32]. HeLa-229 cells, grown in 48-well tissue culture plates, were inoculated with 10-fold serial dilutions of the vaginal swabs and incubated for 40 h at 37 °C. The monolayers were fixed with methanol, and chlamydial IFU were stained using the monoclonal antibody (mAb)-E4 that recognizes the VD-4 in MOMP [33]. The limit of detection was two *C. trachomatis* IFU/culture.

#### 2.2.2. Fertility Studies

At seven weeks following infection, female mice were housed with proven breeder male mice for 18 days (ratio, 4F/1M) [32]. Groups of non-infected mice of the same age as those infected were used as fertility controls. Mice that had an increase in body weight of more than 5 g were considered to be pregnant and were euthanized. Non-pregnant mice were caged for a second time with a male mouse that had fathered during the first cycle of mating. Following euthanasia, the presence of hydrosalpinx was evaluated in situ by exposing the upper genital tract, and the number of embryos in both uterine horns was counted. One C3H/HeN mouse infected with serovar H and one BALB/c mouse from the fertility control group were found dead while caged with the males.

#### 2.2.3. Statistical Analyses

The two-tailed unpaired Student’s *t* test, the Mann–Whitney Rank U test, and the Fisher’s exact test were used for statistical analyses with the program Prism 6.0 (Graphpad). The two groups of mice were compared as described by Rubin [34]. Differences were considered statistically significant for *p* values of <0.05.

## 3. Results

### 3.1. Results of Vaginal Cultures from C3H/HeN Mice Infected with C. trachomatis

Ten groups of mice with eight mice/group were treated with MPA, and four days later, eight groups were infected t.c. with 10^5^ IFU/mouse, each group with one of the eight *C. trachomatis* genitourinary serovars. Two controls, one mock inoculated with SPG and another one not treated (fertility control), were included in this study. Vaginal cultures were collected twice a week for the first four weeks and weekly for two additional weeks. The first culture was collected at day 4 post-infection. The experiment was replicated once.

As shown in Table 1, all C3H/HeN mice except those infected with serovar J had positive vaginal cultures. In comparison with the other groups, the mice infected with serovar J, 56% (9/16), had significantly less positive vaginal cultures (*p* = 0.0008). The control mice inoculated with SPG had negative vaginal cultures.

A total of 160 vaginal cultures were collected from each group of mice (16 mice × 10 cultures) (Table 1). The mice infected with serovar H had the highest percentage of positive vaginal cultures, 77% (123/160), while the group infected with serovar J had the lowest, 8% (12/160) (*p* < 0.0001). No significant differences were found between the combined percentage of positive cultures for serovars from the B-complex (D, E), 49% (156/320), for the B-related complex (F, G), 48% (153/320), for the C-complex (H, I, J), 44% (209/480), and for the C-related complex (K), 46% (74/160) (*p* > 0.05).

The longest time to clearance was observed in the mice infected with serovar H, mean 40 ± 3 days, and the shortest time was observed in the group inoculated with serovar J, 8 ± 1 (*p* < 0.0001) (Table 1). The mean time to clearance for the B-complex (D, E) was 23 ± 1, for the B-related complex (F, G) was 25 ± 2, for the C-complex (H, I, J) was 23 ± 2, and for the C-related complex (K) was 22 ± 2 days (*p* > 0.05).

The mice infected with serovar D had the most severe vaginal shedding (median #IFU/mouse: 37,383; range 5035–296,127), while those inoculated with serovar J had the lowest vaginal shedding (8; range < 2–574) (*p* = 0.0001) (Table 1). The median of the total number of IFU recovered for the B-complex (D, E) was 11,153 (range: 494–296,127), for the B-related complex (F, G) was 862 (range: 12–6402), for the C-complex (H, I, J) was 1819 (<2–14,308), and for the C-related complex (K) was 7930 (range: 1908–86,159). The B-complex shed a significantly higher number of IFU compared with two of the other three complexes, D+E vs. F+G (*p* < 0.0001) and D+E vs. H+I+J (*p* < 0.0001), but not with K (*p* = 0.4546). Complexes F+G vs. K were also significantly different (*p* < 0.0001).

### 3.2. Upper Genital Tract Pathology and Infertility in C3H/HeN Mice Following the Transcervical Infection with the C. trachomatis Serovars

To assess the effects of a *C. trachomatis* infection in upper genital tract pathology and fertility, seven weeks post-infection, C3H/HeN female mice were caged with proven breeder male mice at a ratio of 4/1. Animals that gained more than 5 g of body weight were considered to be pregnant. Pregnant mice were euthanized, the abdominal cavity was open, the genital tract was inspected in situ for the presence of hydrosalpinx, and the number of embryos in each uterine horn was counted. Only the mice infected with serovars D 13% (2/16), H 13% (2/16), I 13% (2/16), and J 6% (1/16) were found to have hydrosalpinxes (Table 1). No significant differences in the number of hydrosalpinxes were found between the eight groups infected with *C. trachomatis* (*p* > 0.05). The fertility control and mice inoculated with SPG had no hydrosalpinxes.

The mice infected with serovar D had the highest fertility rate 75% (12/16), while those infected with serovar K had the lowest 6% (1/16; *p* = 0.0002) (Table 1). Serovars J 13% (2/16; *p* = 0.0010) and I 19% (3/16; *p* = 0.0038) also had lower fertility rates than serovar D. The combined fertility rate for serovars from the B-complex (D, E) was 66% (21/32), for the B-related complex (F, G) was 50% (16/32), for the C-complex (H, I, J) was 23% (11/48), and for the C-related complex (K) was 6% (1/16). Statistically significant differences in fertility rates were found between these complexes [D+E vs. H+I+J (*p* = 0.002); D+E vs. K (*p* = 0.0001); F+G vs. H+I+J (*p* = 0.0163); and F+G vs. K (*p* = 0.0034)]. Both groups of control mice were 100% fertile. All groups of infected mice except for those inoculated with serovar D had a decrease in fertility when compared with the controls (*p* < 0.05).

The mice infected with serovars D or E had the highest average number of embryos per mouse, 5.4 ± 1.0 and 5.3 ± 1.1, respectively, while those infected with serovars J or K had the lowest number, 0.9 ± 0.6 and 0.7 ± 0.7, respectively (Table 1). Differences in the number of embryos were found between serovars D vs. J (*p* = 0.0004); D vs. K (*p* = 0.0004); E vs. K (*p* = 0.0017); and J vs. K (*p* = 0.0019). The average number of embryos for serovars from the B-complex (D, E) was 5.3 ± 0.7, for the B-related complex (F, G) was 3.8 ± 0.7, for the C-complex (H, I, J) was 1.9 ± 0.49, and for the C-related complex (K) was 0.7 ± 0.7 (Table 1). Statistically significant differences were observed between the following complexes: [D+E vs. H+I+J (*p* = 0.0001); D+E vs. K (*p* = 0.0002); F+G vs. H+I+J (*p* = 0.0242); and F+G vs. K (*p* = 0.0080)]. The groups inoculated with SPG and the fertility control had an average of 7.8 and 7.9 embryos/mouse, respectively (*p* > 0.05). All infected mice had significantly less embryos than the fertility controls (*p* < 0.05).

### 3.3. Results of Vaginal Cultures from BALB/c Mice Infected with C. trachomatis

Ten groups of mice with six mice/group were treated with MPA, and four days later, eight groups were infected t.c. with 10^5^ IFU/mouse, each group with one of the eight *C. trachomatis* genitourinary serovars (Table 2). Two negative control groups, one mocked inoculated with SPG and another one not treated (fertility control), were included in the study. Vaginal cultures were collected twice a week for the first four weeks and weekly thereafter. The experiment was replicated once.

Following the t.c. infection with 10^5^ IFU of the *C. trachomatis* genitourinary serovars, 100% of the mice shed except for those inoculated with serovar I 92% (11/12) or J 75% (9/12), but no significant differences were found between the serovars (*p* > 0.05) (Table 2).

A total of 120 vaginal cultures were collected from each group of mice (12 mice × 10 cultures) (Table 2). The mice infected with serovars G or H had the highest number of positive cultures, 51% (61/120), while those infected with serovar J had the lowest number, 22% (26/120; *p* = 0.0001). In comparison to the J serovar, the number of vaginal cultures from the other serovars was significantly different (*p* = 0.0001). No significant differences were found between the combined percentage of positive cultures for serovars from the B-complex (D, E), 38% (91/240), the B-related complex (F, G), 46% (110/240), the C-complex (H, I, J), 38% (138/360), and the C-related complex (K), 34% (41/120) (*p* > 0.05).

Serovar H had the highest mean number of days to clearance, 28 ± 3, while serovar J had the lowest, 20 ± 4 (*p* > 0.05) (Table 2). The mean number of days to clearance for the B-complex (D, E) was 21.7 ± 1.8, for the B-related complex (F, G) was 26.2 ± 2, for the C-complex (H, I, J) was 24 ± 2, and for the C-related complex (K) was 25 ± 3 (*p* > 0.05).

The highest median number of IFU was recovered from the mice infected with serovar D, 13,572 (2400–878,423), while serovar J, 7 (<2–21,995 (*p* = 0.0016), had the lowest number (Table 2). Based on the median number of IFU/complex, statistically significant differences were found only between the B (D+E), 3343 (58–878,423), and the C (H+I+J), 2402 (<2–21,995), complexes (*p* = 0.0409). Approaching a significant difference was observed between the B (D+E) and the B-related (F, G) 1899 (18–53,682) complexes (*p* = 0.0514).

### 3.4. Upper Genital Tract Pathology and Infertility in BALB/c Mice Following the Transcervical Infection with the C. trachomatis Serovars

At seven weeks following the infection, female mice were caged with proven male breeder mice, and the course of the pregnancy was followed by determining the body weight of the females. Mice that gained weight were euthanized, the peritoneal cavity was opened, and the presence of hydrosalpinxes was determined in situ. Only the mice infected with serovars D, 17% (2/12), F, 8% (1/12), and H, 8% (1/12), developed hydrosalpinxes (Table 2). No significant differences in the number of hydrosalpinxes were found between the eight groups infected with the *C. trachomatis* serovars (*p* > 0.05). None of the control mice had hydrosalpinx.

The mice infected with serovars G or J had the highest fertility rates, 92% (11/12), while the lowest fertility rate, 67% (8/12), was found in the mice inoculated with serovar I or K (*p* > 0.05) (Table 2). The combined fertility rates for serovars from the B-complex (D, E), 79% (19/24), the B-related complex (F, G), 88% (21/24), the C-complex (H, I, J), 78% (28/36), and the C-related complex (K), 67% (8/12), were not significantly different (*p* > 0.05). The fertility rates for the SPG-inoculated control and the fertility control were 75% (9/12) and 82% (9/11), respectively (*p* > 0.05), which were not significantly different from the infected groups (*p* > 0.05).

The mice infected with serovars F or H had the highest mean number of embryos, 6.8 per mouse, while those infected with serovar D had the lowest, 4.8 (*p* > 0.05) (Table 2). The average number of embryos for serovars from the B-complex (D, E) was 5.4 ± 0.5, for the B-related complex (F, G) was 6.6 ± 0.6, for the C-complex (H, I, J) was 6.1 ± 0.6, and for the C-related complex (K) was 6.1 ± 1.0 (*p* > 0.05). The SPG mock-infected control and the fertility control had 5.3 ± 0.7 and 5.6 ± 1.1 embryos per mouse, respectively (*p* > 0.05), which were not significantly different from the infected groups (*p* > 0.05).

## 4. Discussion

The goals of this study were to determine, in C3H/HeN and BALB/c mice, the ability of the eight human *C. trachomatis* genitourinary serovars to infect and produce upper genital tract pathology and infertility following a transcervical inoculation. Vaginal cultures showed that the number of *C. trachomatis* IFUs recovered was higher in the C3H/HeN mice inoculated with serovars from the B-complex (D and E) than from the C-complex (H, I, J) and the B- (F and G) and the C-related complexes (K). The C3H/HeN mice inoculated with serovars from the C- or the B-related complexes had more infertility than those inoculated with serovars from the B-complex. No consistent differences in vaginal shedding or infertility were observed in the BALB/c mice inoculated with the eight serovars. The results in the C3H/HeN mice support the findings in humans that serovars from the B-complex establish the most productive vaginal shedding and that *C. trachomatis* serovars from the C-complex induce more upper genital tract pathology resulting in infertility than those from the B-complex. The findings in the BALB/c mice, on the other hand, back human studies that reported no differences in the pathogenicity among the *C. trachomatis* serovars.

Pioneering work by Tuffrey and Taylor-Robinson [35] demonstrated that *C. trachomatis* could not infect mice unless they were pretreated with MPA. Even in mice pretreated with MPA, vaginal inoculation with *C. trachomatis* did not established infection or induce upper genital tract pathology. However, in certain strains of mice, inoculation in the uterine horn or in the ovarian bursa resulted in acute salpingitis, followed by hydrosalpinx formation and infertility [36,37]. Recently, Gondek et al. [31] showed that transcervical infection of MPA pre-treated C57BL/6 mice with *C. trachomatis* L2 led to upper genital tract pathology. Here, we chose to use this infection model since it more closely resembles the route of human infection than the uterine horn or the ovarian bursa inoculation. The study by Gondek et al. [31] utilized C57Bl/6 mice and the serovar L2 that, in humans, produces lymphogranuloma venereum, a disease quite different from those resulting by infections with the oculo-genital serovars. Therefore, the findings by Gondek et al. [31] in C57Bl/6 mice may not be applicable to humans infected with those serovars. For that reason, we decided to use C3H/HeN and BALBc mice that are known to be more susceptible to infection by *C. muridarum* than C57Bl/c mice [27,28]. The long-term goal of this study is to provide the information we need with the *C. trachomatis* oculo-genital serovars so that we can test the efficacy of vaccines against them. C57Bl/6 mice have a strong Th1-biased response, and therefore, humans with similar immune responses likely will not greatly benefit from vaccination, while humans with immune responses similar to those of BALB/c or C3H/HeN mice will [38].

To compare the virulence of the *C. trachomatis* serovars, Ito et al. [39] treated CF-1 mice with MPA and infected them vaginally with 4.8 × 10^7^ to 9.5 × 10^7^ IFUs of serovars D, E, F, G, H, I, or K. Based on the duration of the vaginal shedding, they concluded that serovars D and E were the most virulent and proposed that the increased amount of shedding found in mice infected with these two serovars, as a result of the increased transmission, could explain their higher prevalence in the human population. Lyons et al. [40] isolated four strains of serovar E from symptomatic or asymptomatic females and infected CF-1 mice with these isolates. No differences in the incidence or duration of the infection or upper genital tract pathology were found between the four strains. Based on these results, they concluded that host factors play a major role in the pathogenesis of *C. trachomatis* infections [40].

Our results also showed that serovars D and E shed more than other serovars, confirming the data published by Ito et al. [39] and supporting their suggestion that this may explain the higher prevalence of these serovars in humans. Interestingly, in the C3H/HeN mice, the amount of vaginal shedding did not correlate with upper genital tract pathology. Serovars from the C-complex shed less but induced more infertility than serovars from the B-complex. These results indicate that the ability of a particular serovar to replicate does not necessarily correlate with the long-term sequelae it can induce, including infertility. The lack of correlation between vaginal shedding and upper genital tract pathology has previously been reported with *C. muridarum* and *C. trachomatis* [41,42].

Millman et al. [43] analyzed the DNA sequence of MOMP from 27 *C. trachomatis* isolates, including those tested in this study, and concluded that there is a higher degree of recombination in the C-complex than in the B-complex. The highest degree of recombination and breakpoints was in the VD3 and VD4 regions of MOMP that are surface exposed and, therefore, subject to antibody-mediated immunological pressure. Based on these findings, they proposed that the serovar-specific mutations in MOMP may account for differences in immune surveillance and persistence. The increased rate of mutations in MOMP from the C-complex serovars could result in more robust local immune responses, leading to increases in pathology. Also, based on the breath of cross-reactivity, serovars D and E are evolutionarily seniors to serovars from the C-complex [7]. Although this relationship is not yet well characterized, it could also explain the better adaptation of humans to serovars from the B-complex than to the other complexes [8]. These results imply that MOMP is a potential virulence factor.

In addition to MOMP, there are other potential virulence factors in *C. trachomatis* that have been proposed to affect the outcome of an infection, among others, proteins from the type III secretion system, the inclusion membrane proteins, and the polymorphic outer membrane proteins [44,45,46]. However, none of these proteins have yet been shown to be associated with increases or decreases in upper genital tract pathology either in humans or mice. Although the DNA sequence of MOMP has been found to be quite stable, there are strains from some serovars than can be more pathogenic than others, and therefore, other proteins likely contribute to the virulence of *C. trachomatis* [30].

Due to the different immunogenetic backgrounds of each individual and the diversity of microorganisms, interactions and outcomes between humans and pathogens are difficult to predict. Resistance and susceptibility to *C. trachomatis* infections may be influenced by multiple host genotypic factors, including mutations in toll-like receptors, mannose-binding lectin, chemokine receptors, cytokines, and human leukocyte antigen (HLA) [44,45,46,47,48]. For example, among HLA genotypes, Kinnunen et al. [47] correlated *C. trachomatis*-induced tubal pathology with the *HLA*-DQA1*0102 and the *HLA*-DQB1*0602 alleles. Also, Ohman et al. [48] associated the IL-10-1082AA and the TNF-α-308 promoter alleles with increased tubal infertility.

To evaluate the role of the immunogenetic background of the host to respond to an infection with the *C. trachomatis* serovars, we tested the same dose and route of infection in two strains of mice. Following a vaginal challenge with *C. muridarum,* dissimilarities in susceptibility to infection and infertility were reported between the BALB/c and C3H/HeN mice [27,28]. The BALB/c mice were more resistant to infection and infertility than C3H/HeN mice induced by *C. muridarum*. The results obtained here also demonstrate that C3H/HeN mice are more susceptible than BALB/c mice to a *C. trachomatis* genital infection and upper genital tract pathology and emphasize the need to study the immunogenetic background of the host if we want to better understand the pathogenesis of chlamydial infections in the human population.

In humans, Poston et al. [49] characterized the cervical cytokines associated with *C. trachomatis* and found that Th1 cytokines were correlated with protection against ascension and reinfection, while cytokines involved in humoral, type I interferon and Th17 responses were connected with susceptibility. These findings are supported by the results in mice. C57Bl/6 have Th1-biased immune responses in comparison to BALB/c and C3H/HeN mice that have Th2-biased responses, and this corresponds to susceptibility not only to *C. muridarum* [27,28] but also to *Mycobacterium tuberculosis*, another intracellular bacterium [38]. The level of Th2 immune responses in C3H/HeN and BALB/c mice has been found to be dependent on the pathogen used for infection [38,50,51,52,53].

An interesting finding is the apparent lack of correlation between hydrosalpinx formation and fertility in C3H/HeN mice infected with the *C. trachomatis* serovars. In contrast, there is usually a good correspondence between hydrosalpinx and infertility when mice are infected with *C. muridarum* [54]. Several factors can account for these differences. Most of the studies performed with *C. muridarum* used the vaginal or the intrabursal route to infect mice, while here, the transcervical route was used. Differences in the infectious dose could be another reason. For example, Vicetti-Miguel et al. [55] observed upper genital tract pathology in mice only after multiple infections with *C. trachomatis* serovar D (UW-3/Cx). Interestingly, Tuffrey et al. [56] infected MPA-treated C3H mice with 5 × 10^5^ IFU of serovar E (strain NI.1) in the uterine horn or intrabursally and evaluated egg transport. Ovulation was induced by giving the mice pituitary gonadotropins before they were euthanized, and the oviducts were inspected for the presence of eggs. From mice infected in the ovarian bursae, they failed to recover eggs during the 260 days of the experiment. Following inoculation in the uterine horn, eggs were recovered only for the first two weeks. Egg transport correlated with ciliary movement in the fimbriae and in the ampullary regions of the oviduct, indicating that failure of the ciliary activity alone could result in infertility. Ultrastructural studies demonstrated that tubal edema, inflammatory cells, mucosal congestion, mucous in the lumen, and loss or disorganized ciliated epithelia also lead to infertility. Microscopic hydrosalpinxes were observed in some specimens. Probably, a similar situation occurred in our studies. The lumen of the tube likely was obstructed, the ciliated epithelial cells were damaged, and, if hydrosalpinxes were present, they could not be detected by macroscopic examination. In humans, similar findings have been reported [45,57,58,59].

A Phase I Clinical Trial of a *C. trachomatis* vaccine has recently been successfully completed [60]. This vaccine, termed CTH522, is a recombinant construct containing most of the MOMP serovar D sequence and the VD4 from serovars D, E, F, and G. If found effective, this vaccine could have a significant impact on the prevalence of *C. trachomatis* infections since it includes antigenic regions for a majority of the serovars that infect humans. However, it will be important to monitor the effectiveness of this vaccine at minimizing infection and preventing infertility in individuals infected by *C. trachomatis* serovars from the C-complex.

A limitation of this study is the use of the eight *C. trachomatis* strains from ATCC rather than fresh clinical isolates. The ATCC isolates are readily available to all investigators and are the most frequently used in experimental models. All these strains were collected between the 1950s and the 1970s. The UW-Cx isolates were recovered from the cervix, the UW-Ur isolates were collected from the male urethra, E was cultured from an individual with active trachoma, and F was found in a baby with inclusion conjunctivitis. Although Ito et al. [39] did not specify the strains used for their experiments, we should assume that they were the same as those utilized in this study. Whether or not these particular strains are representative of those currently present in the human population is unknown.

Another shortcoming of this study is that we infected 10–11-year-old mice, an age when they should be sexually fully mature. The highest number of *C. trachomatis* infections are reported in young individuals between 15 and 20 years of age [1]. The high number of infections at this age may be the result of several factors, including high sexual activity and immaturity of the immune and hormonal systems and the genitourinary tract. Older mice are also more resistant to infection than younger animals [28]. Therefore, it is possible that, if we had tested younger or older mice, we may have observed different results.

To conclude, our data support the studies in humans, indicating that *C. trachomatis* serovars from the C-complex are more pathogenic than those from the B-complex. Importantly, the immunogenetic background of the individual can significantly affect the outcome of a *C. trachomatis* infection, as illustrated by the results in the BALB/c mice. It is interesting that both mouse strains infected with serovar J shed the fewest number of IFU, supporting the results by Eckert et al. [61] showing that, among 11,034 male and female patients, the lowest number of IFU was recovered from individuals infected with this serovar. These findings may have clinical implications. Although the treatment for chlamydial infections with antibiotics is well standardized and is effective in most of the patients, it may be important to further investigate if serovars from the C-complex are more frequently associated with negative treatment outcomes and if this requires changes in patient management. Based on the limited vaginal shedding, infections with serovars from the C-complex will be more difficult to diagnose than those with serovars from the B-complex. Failure to treat these patients may increase their risk of developing long-term sequelae. Evaluating the immunogenetic background of the infected patient may help determine if, for a particular individual, the standard treatment needs to be modified [62]. Furthermore, our current diagnostic molecular methods to detect the presence of *C. trachomatis* in clinical specimens do not determine the type of serovar, therefore limiting the ability to implement more effective patient management measures.

## Figures and Tables

**Table 1:** Vaginal shedding, upper genital tract pathology, and fertility results from C3H/HeN mice infected transcervically with 10^5^ IFU of *C. trachomatis* serovars.

*C. trachomatis* Serovar	% +Mice	% +Cultures	Mean (±SE) Days Clear	Median (Range) # IFU Shed/Mouse	% Mice Hydrox	% Mice Fertile	Mean # Embryos/Mouse
*Ct*-D (UW-3/Cx)	16/16 (100) ^a,f^	84/160 (53) ^a,f^	23 ± 1 ^b,g^	37,383 (5035–296,127) ^c,h,k^	2/16 (13)	12/16 (75) ^f,i^	5.4 ± 1.0 ^e,g,j^
*Ct*-E (Bour)	16/16 (100) ^a,f^	72/160 (45) ^a,f^	24 ± 2 ^b,g^	4058 (494–29,219) ^c,h^	0/16 (0)	9/16 (56) ^d,f,i^	5.3 ± 1.1 ^e,g,j^
B-complex (*Ct*-D+E)	32/32 (100)	156/320 (49)	23 ± 1	11,531 (494–296,127) ^k,l,o^	2/32 (6)	21/32 (66) ^i,l^	5.3 ± 0.7 ^j,n^
							
*Ct*-F (IC-Cal3)	16/16 (100) ^a,f^	59/160 (37) ^a,f^	22 ± 3 ^b,g^	550 (12–4230) ^c,h,k^	0/16 (0)	8/16 (50) ^d,i^	3.8 ± 1.0 ^e,g,j^
*Ct*-G (UW-57/Cx)	16/16 (100) ^a,f^	94/160 (59) ^a,f,i^	28 ± 1 ^b,g,j^	1320 (176–6402) ^c,h,k^	0/16 (0)	8/16 (50) ^d,i^	3.9 ± 1.0 ^e,g,j^
B-related complex *(Ct*-F+G)	32/32 (100)	153/320 (48)	25 ± 2	862 (12–6402) ^k^	0/32 (0)	16/32 (50) ^i,m^	3.8 ± 0.7 ^j,n^
							
*Ct*-H (UW-43/Cx)	16/16 (100) ^a,f^	123/160 (77) ^a,f,i^	40 ± 3 ^b,g,j^	5614 (888–14,308) ^c,h^	2/15 (13)	6/15 (40) ^d,i^	3.0 ± 1.0 ^e,i^
*Ct*-I (UW-12/Ur)	16/16 (100) ^a,f^	74/160 (46) ^a,f^	22 ± 2 ^b,g^	2103 (292–13,550) ^c,h,k^	2/16 (13)	3/16 (19) ^d^	1.9 ± 0.9 ^e^
*Ct*-J (UW-36/Cx)	9/16 (56) ^a^	12/160 (8) ^a,i^	8 ± 1 ^b,j^	8 (<2–574) ^c,k^	1/16 (6)	2/16 (13) ^d^	0.9 ± 0.6 ^e^
C-complex *(Ct*-H+I+J)	41/48 (85)	209/480 (44)	23 ± 2	1819 (<2–14,308)	5/48 (10)	11/47 (23)	1.9 ± 0.5
							
*Ct*-K (UW-31/Cx) (C-related complex)	16/16 (100) ^a,f^	74/160 (46) ^a,f^	22 ± 2 ^b,g^	7930 (1908–86,159) ^c,h^	0/16 (0)	1/16 (6) ^d^	0.7 ± 0.7 ^e^
							
SPG	0/16 (0)	0/160 (0)	0 ± 0	<2	0/16 (0)	16/16 (100)	7.8 ± 0.5
Fertility Control	NT	NT	NT	NT	0/16 (0)	16/16 (100)	7.9 ± 0.4

NT = Not tested. ^a^ Significant (*p* < 0.05) by the Fisher’s exact test compared to the control group inoculated with SPG. ^b^ Significant (*p* < 0.05) by the Student’s *t* test compared to the control group inoculated with SPG. ^c^ Significant (*p* < 0.05) by the Mann–Whitney U test compared to the control group inoculated with SPG. ^d^ Significant (*p* < 0.05) by the Fisher’s exact test compared to the fertility control group. ^e^ Significant (*p* < 0.05) by the Student’s *t* test compared to the fertility control group. ^f^ Significant (*p* < 0.05) by the Fisher’s exact test compared to the group inoculated with serovar J. ^g^ Significant (*p* < 0.05) by the Student’s *t* test compared to the group inoculated with serovar J. ^h^ Significant (*p* < 0.05) by the Mann–Whitney U test compared to the group inoculated with serovar J. ^i^ Significant (*p* < 0.05) by the Fisher’s exact test compared to the group inoculated with serovar K (C-related complex). ^j^ Significant (*p* < 0.05) by the Student’s *t* test compared to the group inoculated with serovar K (C-related complex). ^k^ Significant (*p* < 0.05) by the Mann–Whitney U test compared to the group inoculated with serovar K (C-related complex). ^l^ Significant (*p* < 0.05) by the Mann–Whitney U test compared to the B-related complex (F+G). ^m^ Significant (*p* < 0.05) by the Fisher’s exact test compared to the C-complex (H+I+J). ^n^ Significant (*p* < 0.05) by the Student’s *t* test compared to the C-complex (H+I+J). ^o^ Significant (*p* < 0.05) by the Mann–Whitney U test compared to the C-complex (H+I+J).

**Table 2:** Vaginal shedding, upper genital tract pathology, and fertility results from BALB/c mice infected transcervically with 10^5^ IFU of *C. trachomatis* serovars.

*C. trachomatis* Serovar	% +Mice	% +Cultures	Mean (±SE) Days Clear	Median (Range) # IFU Shed/Mouse	% Mice Hydrox	% Mice Fertile	Mean # Embryos/Mouse
*Ct*-D (UW-3/Cx)	12/12 (100) ^a^	43/120 (36) ^a,d^	22 ± 3 ^b^	13,572 (2400–878,423) ^c,e,g^	2/12 (17)	9/12 (75)	4.8 ± 0.6
*Ct*-E (Bour)	12/12 (100) ^a^	48/120 (40) ^a,d^	21 ± 2 ^b^	2118 (58–3443) ^c,e^	0/12 (0)	10/12 (83)	5.9 ± 0.7
B-complex (*Ct*-D+E)	24/24 (100)	91/240 (38)	21 ± 2	3343 (58–878,423) ^h^	2/24 (8)	19/24 (79)	5.4 ± 0.5
							
*Ct*-F (IC-Cal3)	12/12 (100) ^a^	49/120 (41) ^a,d^	26 ± 2 ^b^	533 (18–36,325) ^c,e^	1/12 (8)	10/12 (83)	6.8 ± 0.8
*Ct*-G (UW-57/Cx)	12/12 (100) ^a^	61/120 (51) ^a,d,f^	26 ± 2 ^b^	2203 (652–53,682) ^c,e^	0/12 (0)	11/12 (92)	6.4 ± 0.7
B-related complex *(Ct*-F+G)	24/24 (100)	110/240 (46)	26 ± 2	1899 (18–53,682)	1/24 (4)	21/24 (88)	6.6 ± 0.6
							
*Ct*-H (UW-43/Cx)	12/12 (100) ^a^	61/120 (51) ^a,d,f^	28 ± 3 ^b^	6421 (116–16,285) ^c,e^	1/12 (8)	9/12 (75)	6.8 ± 1.0
*Ct*-I (UW-12/Ur)	11/12 (92) ^a^	51/120 (42) ^a,d^	23 ± 3 ^b^	2550 (<2–11,064) ^c,e^	0/12 (0)	8/12 (67)	4.9 ± 0.9
*Ct*-J (UW-36/Cx)	9/12 (75) ^a^	26/120 (22) ^a,f^	20 ± 4 ^b^	7 (<2–21,995) ^c,g^	0/12 (0)	11/12 (92)	6.6 ± 0.9
C-complex *(Ct*-H+I+J)	32/36 (89)	138/360 (38)	24 ± 2	2402 (<2–21,995)	1/36 (3)	28/36 (78)	6.1 ± 0.6
							
*Ct*-K (UW-31/Cx) (C-related complex)	12/12 (100) ^a^	41/120 (34) ^a,d^	25 ± 3 ^b^	3216 (733–11,421) ^c,e^	0/12 (0)	8/12 (67)	6.1 ± 1.0
							
SPG	0/12 (0)	0/120 (0)	0 ± 0	<2	0/12 (0)	9/12 (75)	5.3 ± 0.7
Fertility Control	NT	NT	NT	NT	0/11 (0)	9/11 (82)	5.6 ± 1.1

NT = Not tested. ^a^ Significant (*p* < 0.05) by the Fisher’s exact test compared to the control group inoculated with SPG. ^b^ Significant (*p* < 0.05) by the Student’s *t* test compared to the control group inoculated with SPG. ^c^ Significant (*p* < 0.05) by the Mann–Whitney U test compared to the control group inoculated with SPG. ^d^ Significant (*p* < 0.05) by the Fisher’s exact test compared to the group inoculated with serovar J. ^e^ Significant (*p* < 0.05) by the Mann–Whitney U test compared to the group inoculated with serovar J. ^f^ Significant (*p* < 0.05) by the Fisher’s exact test compared to the group inoculated with serovar K (C-related complex). ^g^ Significant (*p* < 0.05) by the Mann–Whitney U test compared to the group inoculated with serovar K (C-related complex). ^h^ Significant (*p* < 0.05) by the Mann–Whitney U test compared to the C-complex (H+I+J).

## Data Availability

The original contributions presented in this study are included in the article. Further inquiries can be directed to the corresponding author.
